# Persistent transmission of malaria in Garo hills of Meghalaya bordering Bangladesh, north-east India

**DOI:** 10.1186/1475-2875-9-263

**Published:** 2010-09-22

**Authors:** Vas Dev, Barlind M Sangma, Aditya P Dash

**Affiliations:** 1 National Institute of Malaria Research (Field Station), Chachal, Guwahati - 781 022, Assam, India; 2 National Vector Borne Disease Control Programme, District Malaria Officer, Tura - 794 001, Meghalaya, India; 3 National Institute of Malaria Research (ICMR), Dwarka, Delhi - 110 077, India

## Abstract

**Background:**

Malaria is endemic in Garo hills of Meghalaya, and death cases are reported annually. *Plasmodium falciparum *is the major parasite, and is solely responsible for each malaria-attributable death case. Garo hills are categorized high-risk for drug-resistant malaria; however, there exists no data on malaria transmitting mosquitoes prevalent in the region. Included in this report are entomological observations with particular reference to vector biology characteristics for devising situation specific intervention strategies for disease transmission reduction.

**Methods:**

The epidemiological data of the West Garo hills have been reviewed retrospectively for 2001-2009 to ascertain the disease transmission profile given the existing interventions. Point prevalence study was conducted in Dalu Community Health Centre that lies in close proximity to international border with Bangladesh to ascertain the true prevalence of malaria, and parasite species. Mosquito collections were made in human dwellings of malaria endemic villages aiming at vector incrimination, and to study relative abundance, resting and feeding preferences, and their present susceptibility status to DDT.

**Results:**

Investigations revealed that the West Garo hill district is co-endemic for *Plasmodium falciparum *and *Plasmodium vivax*, but *P. falciparum *was the predominant infection (> 82%). Malaria transmission was perennial and persistent with seasonal peak during May-July corresponding to months of high rainfall. Entomological collections revealed that *Anopheles minimus *was the predominant species that was incriminated by detection of sporozoites in salivary glands (infection rate 2.27%), and was ascertained to be fully susceptible to DDT.

**Conclusion:**

For the control of malaria, improved diagnosis and sustained supply of drugs for artemisinin-based combination therapy are strongly advocated, which should be enforced for treatment of every single case of *P. falciparum*. Greater political commitment is called for organized vector control operations along border/high-risk areas to contain the spread of drug-resistant malaria, and averting impending disease outbreaks.

## Background

Malaria is major public health concern in the north-eastern states of India that continues to deter the equitable socio-economic development of the region [1]. Among seven sister states of north-east India, much of the research investigations related to malaria epidemiology and control were reported from Assam [2]. Of its adjoining states which are just as malaria endemic, Meghalaya (25° - 26° N latitude & 90° - 93° E longitude) contribute > 20% of cases of those reported from the north-east states annually [3]. Despite full logistics support and increased allocation of resources from centrally funded schemes of National Vector Borne Disease Vector Control Programme (NVBDCP), case incidences are on the rise with attributable death reports associated with focal disease outbreaks that are visiting annually affecting bordering population groups. There exists little data on abundance, infectivity, and feeding behaviour of malaria transmitting mosquito vector species, and to their present susceptibility status to DDT, the commonly used residual insecticide for vector control. Among all districts of Meghalaya that are malaria endemic, the Garo hills persistently contribute the majority of *Plasmodium falciparum *cases (> 60%) and attributable deaths, and the district is categorized high-risk for drug-resistant malaria (source, state health directorate). In understanding the local disease epidemiology for disease prevention and containment, the objectives of the present study were to review the malaria situation retrospectively for the years between 2001 - 2009 under present disease surveillance, and report entomological observations with particular reference to vector biology characteristics for devising situation specific intervention strategies for disease transmission reduction. The study had the approval of the institutional ethics committee.

## Methods

### Study area

**Figure 1:**
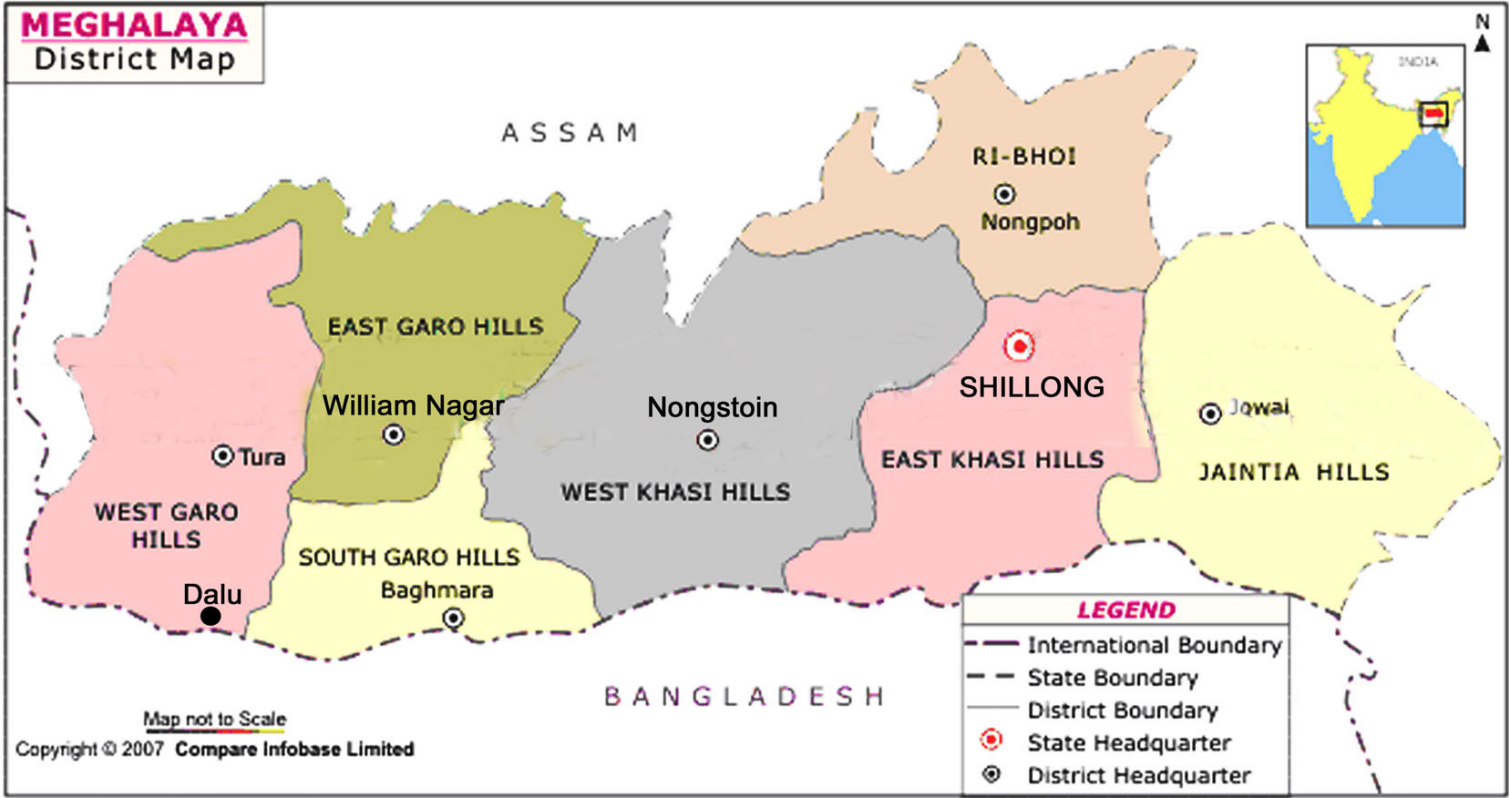
**District map of Meghalaya state showing interstate border with Assam to the North, and international border with Bangladesh to the South**. The symbol (black dot) denotes geographical location of Dalu in the West Garo Hill district close to Bangladesh. Inset is a map of India showing location of the state of Meghalaya.

The investigations were focused in the West Garo Hill district that is prone to focal disease outbreaks characterized by an increase in *P. falciparum *cases and associated deaths ascribed to multi-drug resistant malaria. The district is mostly hilly bounded by interstate border with Assam to the North, and international border with Bangladesh to the South (Figure 1). The terrain is difficult, population is sparse, and tribal aborigines are the majority ethnic population (> 70%), living in rural areas and practicing shifting paddy cultivation for subsistence. It rains heavily during May to September, and the total rainfall varies from 2.5 - 4.5 meters annually. During these months, many of the foothill villages are affected by waves of flash floods limiting access to healthcare services. For most of the year, average temperatures (23 - 28°C) and relative humidity (> 70%) are conducive for mosquito breeding and longevity permitting perennial transmission.

### Disease surveillance and control interventions

In West Garo Hill district, there are 24 Primary Health Centers/Community Health Centers each of which has diagnostic and treatment facilities for malaria that serve as reporting centre for malaria incidence under NVBDCP. The data on malaria incidences from all sources, i.e., active fever surveillance based on fortnightly domiciliary visits, passive case detection (malaria clinic), and mass and contact surveys were pooled monthly/annually. For vector control interventions, annual two rounds of residual spraying of DDT @ 1 gm per square meter were conducted in areas reporting high incidence of *P. falciparum *cases and attributable deaths, of which first round was usually conducted during March - May, and second beginning July to be completed by end of September each year. Other measures included provision for focal spraying (in areas reporting disease outbreaks characterized by high rise in case incidences), impregnation of community-owned mosquito nets, and supply of mosquito net gratis in below poverty population groups. In addition, programmes of health education and awareness for self-protection supported by media and non-governmental organizations were routinely conducted particularly during months of high transmission period. The anti-parasite measures included chemotherapy for every single confirmed cases of malaria by anti-malarial drug policy in force that was subject to revision periodically. Chloroquine was the drug of choice up until 2004, when it was replaced by sulphadoxine-pyrimethamine, and beginning 2007, it was changed over to combination therapy (artesunate + sulphadoxine-pyrimethamine) for treatment of *P. falciparum *cases. Re-orientation and training programmes for health personnel are conducted annually as capacity building exercises for strengthening healthcare services particularly in high-risk areas.

### Entomological techniques

Although malaria cases are reported across the district, there is greater concentration of cases in foothill villages that lie close to inter-state/inter-country borders. Thus, to ascertain the prevalent anopheline mosquito species, daytime resting collections were made by experienced insect collectors in malaria endemic villages of the Dalu Community Health Centre that lie close to the international border with Bangladesh (Figure 1). Mosquitoes adults resting on walls and household articles in human dwellings (indoors) were sampled during early hours (07:00 -09:00 h), and were identified using regional pictorial taxonomical keys to the species level for relative abundance expressed as person hour density. In addition dusk-to-dawn (18:00 - 05:00 h) human landing catches were made in house dwellings (both indoors and outdoors) on the hourly basis to ascertain the biting behaviour of mosquitoes for predilection for human host for which informed consent was taken from the participating volunteers. The mosquitoes collected of both sources were dissected in 0.9% saline for detection of sporozoite infection in salivary glands. The entomologic inoculation rate (EIR) was calculated as the product of human mosquito landing rate and the proportion of mosquitoes carrying sporozoite infection. The mosquito species thus incriminated were subject to insecticide susceptibility test against DDT (4%) using standard WHO diagnostic test kit procedures.

### Parasitological investigations

To ascertain the true disease prevalence and parasite species, besides malaria clinic (passive case detection), active fever surveillance was instituted in villages of Dalu Community Health facility for the duration of the study period (May - June 2007). Subjects reporting fever or with history of fever over past few days (clinical cases) were screened for malaria parasite in peripheral finger prick blood-smear using gold standard microscopic technique. Both thick and thin blood-smears stained with Jaswant Singh Bhattacharya (JSB) stain were examined for at least 100 microscopic fields for malaria parasite and species identification before declaring slide negative. Malaria positive cases were administered anti-malarial drugs as per existing national drug policy in force under medical supervision.

## Results

### Malaria morbidity and seasonal prevalence

**Figure 2:**
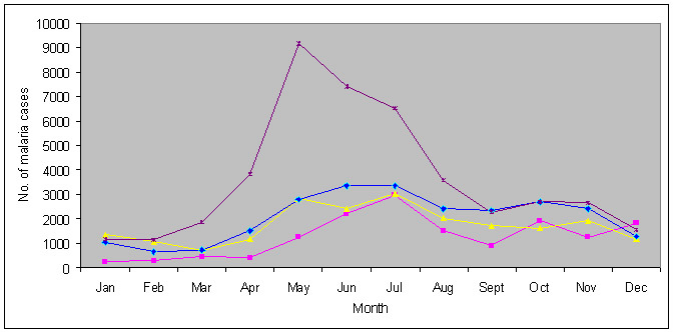
**Monthly distribution of malaria cases in the West Garo hill district of Meghalaya in 2006 (pink square), 2007 (yellow triangle), 2008 (blue spade) and 2009 (violet cross)**.

The available data on malaria-attributable morbidity for the years 2001 - 2009 are presented in Table 1. For each year, the annual blood examination rate (% of population screened) was > 10% (the expected rate of fever prevalence in the communities), and considered adequate as per guidelines of the National Vector Borne Disease Control Programme. The overall smear parasite rate (SPR) for the year ranged from 6.4% in 2002 to 18.8% in 2009. Similar trends were seen for annual parasite incidences (API) that varied from 12.9 to 68.3 confirmed cases per thousand population/year. Considered for the given year, of total cases confirmed positive, *P. falciparum *was the predominant infection (> 98%), all others were *Plasmodium vivax *cases. Malaria cases were recorded in all months, however, with the commencement of the rainy season in April, there was sudden rise in cases beginning May that peaked during May - July (Table 2). These were also the months of high rainfall. Beginning August, there was steady decline in cases but numbers varied with record low during January - April corresponding to dry months/low rainfall. The transmission profile was quite consistent for the years reported but the intensities varied (Figure 2). The cases registered were the highest in 2009, but *P. falciparum *was the major parasite for all months and years together. Death cases confirmed to be due *P. falciparum *malaria were recorded each year, and varied from 15 in 2001 to 98 in 2007, and comprised all age-groups of both sexes equally (*x^2 ^*= 0.111, *P *= 0.70). The point prevalence study in Dalu Community Health Centre of the district that lies in close proximity to Bangladesh border revealed that malaria was common ailment in all age groups, and overall parasite rate was 18.2 per cent (207/1136) among those reporting fever (Table 3). Of total confirmed positive cases by microscopic examination of blood-smear, *P. falciparum *constituted 82.6%, and the remaining were *P. vivax *cases.

**Table 1:** Malaria-attributable morbidity in the West Garo hill district of Meghalaya, India*

Year	Population	No. and (%) of blood-smears			% of positive blood-smears with *P. falciparum*	Annual parasite incidence (no. of cases per 1000 population)	Annual blood examination rate (% of population checked)	Death cases
						
		Examined	Positive for malaria parasite (parasite rate)	Positive for *P. falciparum*				
2001	537346	103371	8053 (7.7)	7971	98.9	14.9	19.2	15

2002	550691	110371	7108 (6.4)	7075	99.5	12.9	20.0	21

2003	563120	87753	8910 (10.1)	8862	99.5	15.8	15.5	25

2004	570492	106095	9646 (9.0)	9568	99.1	16.9	18.5	26

2005	574478	92743	8583 (9.2)	8533	99.4	14.9	16.1	20

2006	578683	131449	15203 (11.5)	15091	99.2	26.2	22.7	36

2007	606062	151639	21057 (13.8)	20914	99.3	34.7	25.0	98

2008	630068	160675	23757 (14.7)	23658	99.5	37.7	25.5	31

2009	642139	232318	43879 (18.8)	43694	99.5	68.3	36.1	67

*Source, State Health Directorate of Meghalaya

**Table 2:** Meteorological data and monthly distribution of malaria cases in the West Garo hill district of Meghalaya, India (Source, State Health Directorate of Meghalaya)

Month (2006)	Meteorological data					No. of blood-smears examined	No. (%) of blood-smears		Monthly parasite incidence (no. of cases per 1000 population)	Death cases
					
	Rain fall (mm)	No. of rainy days	Temperature (°C)		Average Relative Humidity (%RH)		Positive for *P. falciparum*	Positive for *P. vivax*		
									
			Max	Min						
January	0	0	28.0	10.0	65	3891	256 (6.6)	01 (0.03)	0.44	0

February	26.8	3	35.1	20.2	65	4586	272 (5.9)	0 (0)	0.47	0

March	12.2	2	35.0	20.0	58	6097	452 (7.4)	08 (0.1)	0.79	0

April	204.6	9	27.7	25.0	67	5324	420 (7.9)	10 (0.2)	0.74	3

May	708.2	19	27.4	24.0	72	10108	1207 (11.9)	23 (0.2)	2.12	8

June	442.8	20	27.0	24.4	76	19484	2192 (11.3)	07 (0.04)	3.80	3

July	463.2	24	28.2	27.0	73	21329	2957 (13.9)	12 (0.06)	5.13	5

August	246.2	20	29.2	26.0	74	11623	1500 (12.9)	03 (0.03)	2.59	2

September	318.4	19	28.2	25.1	75	9913	886 (8.9)	0 (0)	1.53	0

October	189.8	10	29.0	25.9	68	15867	1929 (12.2)	10 (0.06)	3.35	4

November	0	0	26.9	24.1	71	12107	1230 (10.2)	08 (0.07)	2.14	7

December	0	0	24.9	21.8	74	11120	1790 (16.1)	30 (0.3)	3.14	4

**Table 3:** Prevalence of malaria in Dalu Community Health Centre, West Garo hill district of Meghalaya, India*

Age group in years	No. of blood- smears examined	No. & (%) of smears positive for malaria	No. & (%) of malaria cases positive for *P. falciparum*
1 - 4	283	58 (20.5)	46 (79.3)

5 - 8	245	51 (20.8)	42 (82.3)

9 -14	205	53 (25.8)	44 (83.0)

≥ 15	403	45 (11.2)	39 (86.7)

**Total**	**1136**	**207 (18.2)**	**171 (82.6)**

*Study period, May - June 2007

### Entomological observations

#### Mosquito abundance, biting behaviour and infectivity

Indoor day-resting collections from human dwellings revealed that *Anopheles aconitus, Anopheles annularis, Anopheles minimus *and *Anopheles vagus *were prevalent, and their densities varied from 0.05 to 3.18 per person/hour (Table 4). Of these, *An. vagus *and *An. minimus *were common catch constituting bulk of mosquito collections. The majority of *An. minimus *collected were fully fed, semi-gravid or gravid, of which 39% were parous. These data were corroborated by indoor and outdoor human landing catches in the dusk-to-dawn mosquito collections (based on one night) in which *An. minimus *was the most predominant anopheline mosquito species.

**Table 4:** Relative abundance and dissection records of anopheline mosquito species from human dwellings (indoor) in malaria endemic villages of Dalu Community Health Centre of the West Garo hill district of Meghalaya, India*

Mosquito species	No. mosquitoes collected	Abdominal condition**				Density per person hour (person hours = 39)	Vector incrimination	
				
		UF	FF	SG	G		No. dissected	Sporozoite Infection rate (%)
*An. aconitus*	02	2	0	0	0	0.05	2	0 (0)

*An. annularis*	02	0	0	0	2	0.05	02	0 (0)

*An. minimus*	124	4	33	48	39	3.18	88	2 (2.27)

An. vagus***	122	-	-	-	-	3.13	77	0 (0)

*Study period: May - June 2007

** UF = Unfed, FF = Fully fed, SG = Semi gravid, G = Gravid

*** Abdominal condition not scored due to zoophilic nature/lesser significance

The mosquito biting rate per person/night was 4 and 2 indoor and outdoor respectively, whereas only single *Anopheles baimaii *(formerly species D of *Anopheles dirus*) was captured indoors. The peak biting activity of *An. minimus *occurred during midnight 00 to 02:00 h. However, *Culex *species that were most abundant mosquito actively searched human host almost throughout the night, and biting rate were 16 and 11 per person/night indoor and outdoors respectively. Of total mosquitoes dissected, *An. minimus *were incriminated by detection of sporozoites in salivary glands, and infection rate was 2.27% (2/88); all other mosquito species were sporozoite negative. Given the data on mosquito biting rate and sporozoite infectivity, the entomologic inoculation rate (a product of sporozoite infection rate × the human mosquito-biting rate) for both indoor and outdoor was 0.090 and 0.045 respectively. The locally collected adult mosquito populations of *An. minimus *when subjected to DDT (4%) susceptibility test (the residual insecticide in use for vector control) resulted in 100% mortality post 24 h exposure, and was observed to be consistent for the two replicates of 15 mosquitoes each, thus ascertained to be fully susceptible at given diagnostic concentration.

## Discussion

In north-east India, disease distribution is geographically restricted but remains entrenched in population groups living in poverty particularly in foothill villages/inter-border areas [4]. The Garo hill districts in Meghalaya persistently contributed most cases and malaria-attributable deaths, and deserve priority for strengthening interventions. The problem is complex along international borders of the northeast due to poor inter-country coordinated vector control interventions, illiteracy, difficult terrain and poor access to healthcare services [5]. The highest death toll of 98 in 2007 was ascribed to focal disease outbreaks along Indo-Bangla border, and every single death was confirmed to be due to *P. falciparum *malaria that is widely prevalent and believed to be multi-drug resistant [6]. Most death cases that were preventable occurred of complications arising due to late reporting and consequent delayed treatment (source, state health directorate). The disease burden due to malaria is estimated to be much higher for many clinical and death cases for which no blood-smear report was available were excluded from the surveillance data [7]. From the point prevalence study, it was revealing that the relative abundance of *P. falciparum *cases was lower (82%) than what is being reported under state surveillance accounting for 99% of the reported cases (Table 1, 3). Seemingly for want of laboratory expertise, many *P. vivax *cases perhaps were misdiagnosed as *P. falciparum *by state technicians amounting to unwarranted use of anti-malarials. The fact that mixed infections of both the prevalent parasite species were not observed underlines that the transmission intensity is low-to-moderate. The unusual high rise in reported cases, however, beginning 2007 (formerly under-reported) could be ascribed to induction of rapid diagnostic kits (RDKs) for on-the-spot diagnosis by female Accredited Social Health Activists (ASHA) workers in door-to-door surveillance. From the presented data, it was evident that disease transmission is not only perennial but persistent calling for concerted efforts in devising appropriate interventions in place for much needed transmission reduction. It is widely believed that these pockets serve as reservoirs of drug-resistant strains that are fast evolving due to mixing of strains owing to population movement across borders with an opportunity to spread through anthropophilic mosquito vectors that are widely prevalent [8].

The present study clearly established that *An. minimus *is the major vector in Garo hills based on detection of sporozoite infection in the salivary glands. The morphologically identified populations of this species complex in north-east have earlier been characterized to be *An. minimus *based on molecular data [9,10]. Even though *An. minimus *mosquitoes were highly susceptible to DDT, but transmission continued uninterrupted largely due to poor quality spray coverages, and high refusal rates by the communities making it inadequate intervention. The local agricultural practice of shifting cultivation made it difficult for effective surveillance during the period (May - September) which somehow coincided with the high transmission period. This practice presumably resulted in parasite reservoir for many individuals remained untreated rendering them asymptomatic carriers. In north-eastern states, 10-30 per cent of malaria endemic communities are reported to be asymptomatic cases, which are unlikely to seek treatment [11]. Apparently, both the inadequate interventions against vector mosquito populations and parasite reservoir in the communities facilitated the year-round transmission observed. In the adjoining state of Assam with similar transmission dynamics, risk factor is assessed to be much greater in foothill/inter-border villages for infective mosquito bites calling far greater allocation of resources for effective control [12]. Among other documented vector species in the northeast, of *An. dirus *species complex, the role *An. baimaii *(populations of which have been characterized by molecular evidence) could not be clearly substantiated in this locality [13]. Seemingly, its numbers are dwindling due to deforestation at the expense of population explosion and increased acreage under paddy cultivation. Given the entomologic inoculation rates, the transmission intensity is estimated to be low-to-moderate. However, there is every risk of replacement of *P. vivax *with that of drug-resistant *P. falciparum *as that has been reported in central India in similar physiographic locales [14].

It is satisfying that the given the present scenario, interventions are currently being strengthened by increased allocation under Global Fund to fight against AIDS, Tuberculosis and Malaria (GFATM) by additional tools, i.e., the impregnation of community-owned mosquito nets, provision of mosquito net especially for below poverty line families, wide access to effective chemotherapy using artemisinin-based combination therapy (ACT), improved disease surveillance and diagnosis using RDKs, induction of ASHA female workers for early diagnosis, increased involvement of Non Governmental Organizations and civic societies. Aiming at containment of drug-resistant malaria prevalent in Garo hills, we strongly advocate sustained interventions ensuring supply of ACT which should be enforced for treatment of every single case of *P. falciparum*. The therapeutic response to ACT presently in force (artesunate + sulphadoxine-pyrimethamine) has been assessed to be adequate (unpublished observations), and holds good promise in rolling back malaria, and saving lives [15-17]. To avert the malaria disaster, strengthening of healthcare services in the periphery where there is need most for better case management, enhanced DDT spray coverage by making it a community-based activity, increased awareness on disease prevention, and greater political commitment for organized control operations along border/high-risk areas are strongly recommended. It is believed that in areas with low-to-moderate transmission, rolling back malaria is possible with all these combined measures that would help restore confidence in the communities and all round socio-economic development [18,19].

## Conclusion

The Garo hills of Meghalaya that share an international border with Bangladesh are co-endemic for *P. vivax *and *P. falciparum *malaria. Transmission of the causative parasites is perennial and persistent with seasonal peak during May - July corresponding to months of high rainfall. Entomological collections revealed that *An. minimus *was the predominant mosquito species that was incriminated by detection of sporozoites in salivary glands (infection rate 2.27%), and ascertained to be fully susceptible to DDT. For the control of malaria, along with greater political commitment for organized vector control operations along border/high-risk areas, improved diagnosis and sustained supply of artemisinin-based combination therapy are advocated for treatment of every single case of *P. falciparum *averting spread of drug-resistant malaria and impending disease outbreaks.

## Competing interests

The authors declare that they have no competing interests.

## Authors' contributions

VD: Collected and analyzed the data, and developed the first draft of the manuscript. BMS: Epidemiological data collection and compilation, data analysis (in part), and local study coordination. APD: Study planning and coordination. All authors read and approved the final version of the manuscript.
